# Bioinformatics Pipelines for Targeted Resequencing and Whole-Exome Sequencing of Human and Mouse Genomes: A Virtual Appliance Approach for Instant Deployment

**DOI:** 10.1371/journal.pone.0095217

**Published:** 2014-04-21

**Authors:** Jason Li, Maria A. Doyle, Isaam Saeed, Stephen Q. Wong, Victoria Mar, David L. Goode, Franco Caramia, Ken Doig, Georgina L. Ryland, Ella R. Thompson, Sally M. Hunter, Saman K. Halgamuge, Jason Ellul, Alexander Dobrovic, Ian G. Campbell, Anthony T. Papenfuss, Grant A. McArthur, Richard W. Tothill

**Affiliations:** 1 Bioinformatics, Peter MacCallum Cancer Centre, East Melbourne, VIC, Australia; 2 Department of Mechanical Engineering, The University of Melbourne, Parkville, VIC, Australia; 3 YourGene Biosciences Australia, Southbank, VIC, Australia; 4 Molecular Pathology Research and Development Laboratory, Peter MacCallum Cancer Centre, East Melbourne, VIC, Australia; 5 Victorian Melanoma Service, Alfred Hospital, Prahran, VIC, Australia; 6 Department of Epidemiology and Preventive Medicine, Monash University, Clayton, VIC, Australia; 7 Sarcoma Genetics and Genomics Laboratory, Peter MacCallum Cancer Centre, East Melbourne, VIC, Australia; 8 Cancer Genetics Laboratory, Peter MacCallum Cancer Centre, East Melbourne, VIC, Australia; 9 Bioinformatics division, The Walter and Eliza Hall Institute for Medical Research, Parkville, VIC, Australia; 10 Sir Peter MacCallum Department of Oncology, The University of Melbourne, Parkville, VIC, Australia; 11 Molecular Oncology Laboratory, Oncogenic Signaling and Growth Control Program, Peter MacCallum Cancer Centre, East Melbourne, VIC, Australia; 12 Translational Research Laboratory, Cancer Therapeutics Program, Peter MacCallum Cancer Centre, East Melbourne, VIC, Australia; 13 Department of Medicine, St. Vincent’s Hospital, Fitzroy, VIC, Australia; 14 Department of Pathology, University of Melbourne, Parkville, VIC, Australia; 15 Bioinformatics and Cancer Genomics Laboratory, Peter MacCallum Cancer Centre, East Melbourne, VIC, Australia; 16 Translational Genomics & Epigenomics Laboratory, Ludwig Institute for Cancer Research, Heidelberg, VIC, Australia; University of Torino, Italy

## Abstract

Targeted resequencing by massively parallel sequencing has become an effective and affordable way to survey small to large portions of the genome for genetic variation. Despite the rapid development in open source software for analysis of such data, the practical implementation of these tools through construction of sequencing analysis pipelines still remains a challenging and laborious activity, and a major hurdle for many small research and clinical laboratories. We developed TREVA (Targeted REsequencing Virtual Appliance), making pre-built pipelines immediately available as a virtual appliance. Based on virtual machine technologies, TREVA is a solution for rapid and efficient deployment of complex bioinformatics pipelines to laboratories of all sizes, enabling reproducible results. The analyses that are supported in TREVA include: somatic and germline single-nucleotide and insertion/deletion variant calling, copy number analysis, and cohort-based analyses such as pathway and significantly mutated genes analyses. TREVA is flexible and easy to use, and can be customised by Linux-based extensions if required. TREVA can also be deployed on the cloud (cloud computing), enabling instant access without investment overheads for additional hardware. TREVA is available at http://bioinformatics.petermac.org/treva/.

## Introduction

Targeted resequencing (TR) by massively parallel sequencing, which includes whole-exome sequencing (WES), is a well-established and cost-effective means to analyse specific regions of a genome. Previous studies on genetic diversity (e.g. the 1000 genomes project [1]) and on human diseases [2]–[4] have benefited greatly from this sequencing technology. Moreover, with reducing costs of sequencing, TR technologies are becoming an increasingly attractive and feasible option for smaller research groups and clinical laboratories to undertake sequencing projects. Coupled with the popularity of TR is the deluge of bioinformatics tools that have been developed to analyse sequence data, with over 570 tools published within a span of only 2 years [5]. These methods include: FastQC (http://www.bioinformatics.bbsrc.ac.uk/projects/fastqc) and htSeqTools [6] for assessing the quality of short-read data; BWA [7] and Bowtie2 [8] for sequence alignment; MuTect [9] and GATK [10] for detecting single-nucleotide variations; CONTRA [11] and ExomeCNV [12] for identifying copy number aberrations; Genome MuSiC [13] and MutSig (https://confluence.broadinstitute.org/display/CGATools/MutSig) for conducting pathway analysis; and, TREAT [14] and VarSifter [15] for annotation and visualization. Some of these methods are specifically tailored to TR data (e.g. CONTRA, ExomeCNV and TREAT), while others are also applicable to sequence data generated by other technologies. Many of these tools are constantly being improved and updated, with new versions released on a frequent basis (e.g. BWA and GATK).

Despite the large number of tools, the bottleneck in a typical sequencing project remains in the bioinformatics analysis phase due to lack of access to informatics expertise. This is especially the case when a more complex approach that combines multiple tools is required to generate meaningful results for a given study. Without the ability to effectively analyse data, TR technologies are largely under-utilised by many laboratories.

While the larger institutes or laboratories have invested in building data analysis pipelines, many of these pipelines are not transferrable to other labs due to an obfuscated set of dependencies, operating system (OS) incompatibility or porting issues, as well as hardware incompatibility. It is critical that these factors be addressed when developing and distributing a robust pipeline for TR data analysis to ensure that it can be easily adopted by a wide range of researchers or clinicians.

Therefore, rather than conventionally packaging and distributing our pipelines as independent executables/packages/scripts (with ports to different operating systems), we have utilised the concept of a Virtual Machine (VM) to distribute our pipelines in their native OS, alleviating the need to configure and manage both hardware and software dependencies and requirements. The use of a VM to package a complex bioinformatics pipeline is becoming an increasingly attractive alternative to distribute analysis methods that are easily reproduced between laboratories. Our proposed VM, referred to as TREVA (Target REsequencing Virtual Appliance), enables small laboratories and research groups to tap into the vast potential of TR technologies by providing streamlined and rigorously tested pipelines in a convenient and easy-to-use form. We have included a primary and secondary analysis pipeline in TREVA to explore and investigate variations in individual as well as related groups of samples. Both pipelines have been tested and used for the analysis of cancer genomes (published studies include [2], [16]–[18]), and are generally applicable to any human and mouse TR projects. As such, TREVA can also be used as a backbone to build more complex or specialised pipelines.

### Analysis Pipelines for Targeted Resequencing Data

An analysis pipeline in the context of bioinformatics refers to a modular set of tools that are arranged in series, enabling the automation of complex analyses to be conducted on sequence data. Streamlined bioinformatics pipelines for TR/WES are essential since most of these projects involve a constantly changing group of samples, where extra samples can become available for unforeseen reasons, existing samples can become unusable due to technical reasons, and clinical annotation data can be changed or added depending on pathology reviews. Any change would require a re-run of the entire analysis; a well-established pipeline can facilitate data restructuring and reanalyses, and help to avoid repetitive programming.

Although many tools and independent analysis methods are currently available, the development of a pipeline that makes use of these tools/methods is still faced with a large number of challenges. These challenges can be broadly classified into 3 primary areas: design, implementation and bioinformatics expertise.

#### 1. Pipeline design

A typical TR analysis pipeline includes modules that call and interpret single-nucleotide variants (SNVs), short insertion-deletions (INDELs) and exon-level copy number variants (CNVs) for individual samples; and finds significantly mutated genes and pathways among cohorts of samples. The design of each analysis module involves the identification of candidate methods or software packages, which then require testing and evaluation using representative datasets. This is a non-trivial task given the large number of software tools that are publicly available [19]. Similar concerns exist for selecting the correct annotation databases and visualization tools.

#### 2. Pipeline implementation

An extensive, and often prohibitive, amount of time and effort is required to create a ready-to-use pipeline. The laborious tasks during implementation include, but are not limited to, installation and configuration of the various analysis packages, parameter tuning, performance optimisation, input/output interfacing, debugging, and streamlining [20].

#### 3. Bioinformatics expertise

A broad range of highly specialised skills is required to put together an effective and efficient analysis pipeline. From a computational standpoint, special attention needs to be placed on the management of data storage and compute units due to the high-volume of data generated by TR technologies. Operating systems also need to be administered in a way that optimizes efficiency for the bioinformatics algorithms, since they often perform intensive input/output (I/O) operations. From an informatics perspective, a good knowledge of the analysis algorithms is required in order to maintain information integrity. Genomics and biological insights are also critical to the design of a pipeline. These specialised requirements greatly limit the analysis capability of many laboratories [21], especially the smaller clinical laboratories [22].

To tackle some of these challenges, there have been efforts to develop frameworks upon which components of the pipelines can be customised and workflows be defined; examples of these include Taverna [23], Galaxy [24] and Ruffus [25]. However, the implementation and maintenance of these “frameworks” themselves require strong bioinformatics and programming expertise, and users will still face the challenges of pipeline design problem since the frameworks only serve as a blank canvas. Other efforts such as Atlas2 Suite [22] and WEP [26] were designed specifically for whole-exome data. However, they either require strong programming expertise (Atlas2) or require upload to external web servers which associate with storage, bandwidth and security concerns (WEP). Checklist S1 provides a comparison of the features of various pipeline solutions.

As the field of genomics research continues to change rapidly, the time that is available to design and implement a pipeline is very limited for any given analysis problem. Consequently, sophisticated pipelines are often only realized by large sequencing centres, and generally their automated architectures cannot be scaled down or replicated in small to medium sized laboratories or sequencing centres [27].

### The Benefits of a Virtual Appliance

Virtual image technologies (VTs) have been widely adopted in the IT community, and are increasingly gaining popularity. There are several commonly used virtual machines that are free for non-commercial use, such as *VMware Player* or *Oracle VM VirtualBox*. Virtual appliances are ready-to-use, application-focused images built on VTs. They come with a full operating system (OS) and all necessary components pre-configured in the images. Virtual applicances eliminate the need for setting up, testing, debugging, installing, configuring, streamlining, porting to an OS and etc, thereby ultimately minimising the need for specialised computing support. As an example, BitNami (http://bitnami.org/) is an organisation that has made various virtual appliances available in the fields of ecommerce and software project management.

The speed of deployment and efficient maintenance are key drivers of the success of virtual appliances. We see the same needs in bioinformatics, where applications are generally obfuscated in complex layers of dependencies. In a recent publication [28], the use of virtual machines in the context of next-generation sequencing was also discussed and recommended.

By offering our TR/WES analysis pipelines as part of a virtual appliance, users are able to bypass the need for any further setup and can start using the pipelines immediately. Our pipelines are derived from a cancer research centre and can handle a range of data types that are commonly encountered in human disease research. Moreover, packaging our pipelines in a virtual appliance will enable all laboratories, regardless of budget or size, to have access to sophisticated bioinformatics pipelines. Additional knowledge is not required to deploy a virtual appliance (as is the case with Galaxy), and if desired, computer scientists/bioinformaticians can readily extend and build upon the default pipelines through the Linux environment installed in the virtual appliance.

## Results: TREVA – a Targeted REsequencing Virtual Appliance

The pipelines within TREVA are packaged within a fully installed Linux operating system (Ubuntu Luid) on a virtual hard-disk, with all software dependencies already configured. TREVA can be launched on any host platform, and is independent of the software and hardware requirements of the constituent methods in the pipelines. TREVA images are available for download at http://bioinformatics.petermac.org/treva/.

### Analysis Pipelines Included in TREVA

TREVA pipelines cover the detection of genomic variations that are related (but not limited) to cancer studies. We provide a primary and a secondary analysis pipeline. The primary pipeline is used to analyse germline susceptibility or somatic variations (SNVs/INDELs/CNVs), where each sample is considered independently. The secondary pipeline conducts analysis on a cohort of samples taking into consideration any relationships between the samples that are based on predefined clinical or biological grouping of the samples, such as cancer subtype. These pipelines can be run on TR and WES data for human and mouse genomes.

#### Primary analysis pipeline for individual samples

**Figure 1:**
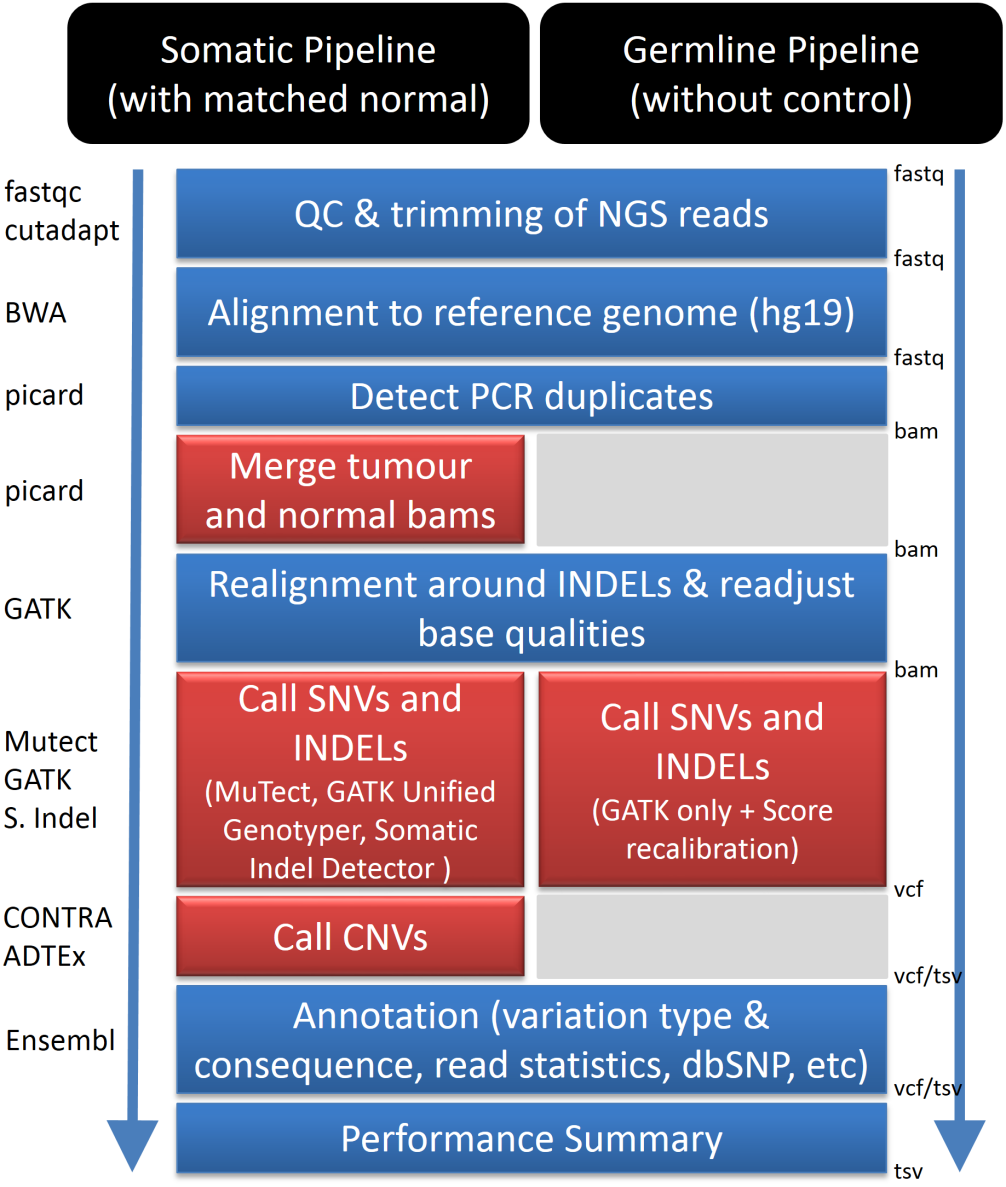
Primary analysis pipelines. Red colour highlights the difference between our Somatic Pipeline and Familial (Germline) Pipeline.

The primary pipeline is outlined in Figure 1. Raw reads (fastq files) are first quality checked with FastQC. Reads that do not pass QC for base qualities are then trimmed using cutadapt [29]. If sequencing adaptors or primers are detected, they are also removed using cutadapt. Filtered reads are then aligned to the appropriate reference genome using BWA [7] and duplicate reads marked using Picard (http://picard.sourceforge.net/). For detection of somatic variants the tumour and normal BAM files are then merged so that GATK INDEL realignment [30] can be performed on both together as per GATK’s best practice recommendations (http://www.broadinstitute.org/gatk/guide/topic?name=best-practices). Base qualities are recalibrated using GATK to correct for inaccurate base qualities [30] to generate the BAM file ready for variant calling.

To identify somatic SNVs and INDELs we use the MuTect [9] and GATK’s Somatic Indel Detector [30] programs, respectively, developed at the Broad Institute. We also use GATK’s Unified Genotyper as a secondary variant caller to assist in identifying true positive somatic variant calls and reducing false positive calls, as we have found a high validation rate for the variants called by both MuTect and GATK. The identified SNVs and INDELs are then combined into a single file and annotated using the Ensembl database. The annotation makes use of a local copy of the Ensembl database and a customised version of Ensembl Variant Effect Predictor [31] (both included with TREVA) to add information such as what gene the variant is in, the consequence of the mutation (nonsynonymous, nonsense, *etc.*) and information from databases such as PolyPhen2 [32], SIFT [33], dbSNP [34], OMIM [35], and COSMIC [36]. We then use CONTRA [11] and ADTEx (http://adtex.sourceforge.net) to analyse copy number variations based on the ratio of read coverage between tumour and normal samples. Custom scripts are used to supplement the output file with additional information from the BAM file corresponding to each variant call, such as: the number of reads that contained the variant, the number of reads that matched the reference, the variant frequency and whether the variant was present in reads that mapped to both forward and reverse strands of the reference (presence on both strands adds confidence to the variant call). The final output of the pipeline consists of a single file containing the annotated variants from the tumour and normal sample.

We use a slightly modified pipeline for calling variants in germline samples for our projects on familial susceptibility to cancer (Figure 1). The distinction between these two pipelines is that only one variant caller is used for germline samples (i.e. GATK’s Unified Genotyper). For sample groups comprising more than one member of a family, all samples are run through the pipeline collectively (i.e. combined into a single BAM file) to improve identification of INDELs and SNVs shared by family members.

#### Secondary analysis pipeline for related groups of samples

**Figure 2:**
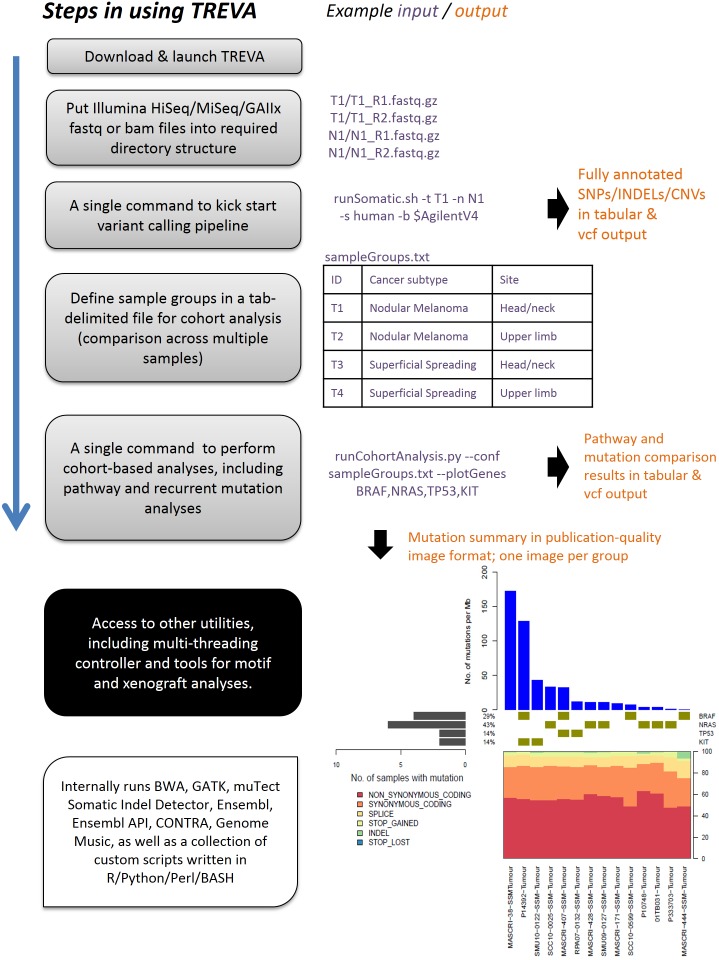
The TREVA workflow: execution of primary and secondary pipelines for variant calling on individual and related groups of samples.

With the lowering cost of TR/WES, experimental designs involving multiple samples are not only feasible but are often utilised to increase the power of a study to detect, for example, driver/recurrent mutations and frequently mutated genes [37]. As an extension to the primary pipeline, our secondary pipeline has been designed to conduct the multi-sample analysis by taking into account any inherent relationships or grouping between samples based on the study design (Figure 2). These relationships are defined by the user in a file and are typically given in terms of clinical annotation. Any additional analysis parameters are also defined in this file. As such, any component of the analysis can be flexibly changed from a single point of reference. This abstraction allows the pipelines to be applied in many different studies without the need to modify or configure any part of the pipelines directly. For instance, contrasts can also be defined between samples if these are of interest.

In the secondary pipeline, the variants called in individual samples by the primary pipeline are first filtered using criteria defined by the user to produce a set of highly confident candidate variants. The output file produced by this step (example available as Table S1) is used for all downstream processing, and can be inspected manually if desired. Nucleotide sequence content is also analysed by first annotating SNVs with flanking nucleotide bases. Summaries of any changes are reported to assist in the interpretation of mutational signatures. A well-documented example of these signatures is the characteristic C to T and CC to TT mutations in melanoma that are representative of UV signatures. Genome MuSiC v0.4 is then used to investigate the presence of significantly mutated pathways, recurrent mutations, clinical correlations and mutation-relations between genes (such as mutual exclusivity of BRAF and NRAS mutations in melanoma). Finally, CNVs that are called by CONTRA and ADTEx are then analysed for recurrent CNVs using GISTIC 2.0 [38], by first converting CONTRA/ADTEx output files into the required GISTIC 2.0 input format.

### The TREVA Workflow

Our proposed workflow for analysing TR/WES data has been designed for ease of use to assist small laboratories in rapidly setting up and executing analysis pipelines with minimal hands-on time or bioinformatics expertise.

#### 1. Launching TREVA

1. TREVA can be launched on a local host using publically available virtual machine software, such as VMware or Oracle VM VirtualBox. After importing the TREVA image, the user will need to set up the data directory such that user data can be seen and processed by the VM. There are no other critical setup requirements to perform, as all the necessary configuration and management of dependencies has already been done.
2. TREVA can also be launched directly from a cloud provider without the need to set up the appliance locally on host machines. In this context, TREVA can be maintained centrally and has the added benefit of scalable computational resources and shared access to all researchers within a laboratory. We have currently made TREVA images available in the Australia’s NeCTAR cloud (National eResearch Collaboration Tools and Resources), which is readily accessible by most Australian academic and research institutes.

#### 2. Setting up input data files

Data files can be made accessible by TREVA by uploading them to the VM/cloud, or by mounting an external file-system containing all the relevant files. Data files can be either BAM files or Illumina HiSeq/MiSeq/GA-IIx fastq files.

#### 3. Running the primary analysis pipeline for individual samples

A single command is all that is required to execute TREVA’s primary variant analysis pipeline. The command takes arguments indicating the sample names for the tumour and normal samples, species (human or mouse), email for progress notification, number of processors, BED file defining the capture assay, and other optional parameters. The resulting output file has a total of 50 columns containing variant annotation and sequence data statistics.

#### 4. Running the secondary analysis pipeline for related groups of samples

Once the samples have been run through the primary pipeline, the secondary analysis pipeline can be executed to analyse the samples in the context of any relationships between them. The user is first required to define the sample groups in a tab-delimited file (the form of the analysis can be conveniently changed by the modifying this file; see example in Table S2). Once this file is prepared, the entire pipeline can be executed by a single command. The analyses that follow include pathway analysis and the identification of significantly mutated genes, which are performed internally using Genome MuSiC; and followed by the invocation of scripts to conduct recurrent mutation analysis and to plot the final results for visualisation (including publication-quality figures).

#### 5. Running additional utilities included in TREVA (optional)

Additional miscellaneous tools have also been included to support parallel processing and other routine tasks. These include a multi-threading controller to manage the processing of a large set of samples in parallel; a tool to detect mouse contamination in sequenced xenograft samples; a tool to conduct motif analysis around single nucleotide variations; a tool to append symbols corresponding overlapping and nearest genes; and a tool to extract the corresponding DNA sequence given as a location in a BED file.

### Case Study – Melanoma Mutational Landscape

In a recently published study [16], TREVA was applied to exome sequence data of 34 fresh frozen primary cutaneous melanomas and matched peripheral blood, with an aim to characterise mutations in melanomas and correlate them with clinico-pathologic features. The entire analysis workflow is summarised as follows:

1. Exome sequencing data was generated using an Illumina HiSeq 2000 on 34 fresh frozen melanoma tumours and matched blood (68 samples in total). Exome capture was performed using either NimbleGen EzExome V2 or Agilent SureSelect Exome V2 capture kits.
2. Two gzipped fastq files containing paired short read data were obtained for each of the 68 samples, and were placed into directories with names corresponding to the sample identifier.
3. Variant calling pipeline (runSomatic.sh in TREVA) was applied to each sample. All samples were processed at once using a batch controller script that comes with TREVA (cmdqueue) to limit the number of concurrent tasks.
4. A tabular file defining the sample groups and clinical variables was prepared by the researcher in Excel. Clinical variables in this study included tumour anatomical site, tumour thickness, tumour subtype, solar elastosis score, pigmentation scores and BRAF/NRAS mutation status (known oncogenic drivers in melanoma).
5. Cohort analysis pipeline (runCohort.py in TREVA) was then applied on the sample definition file. The script matched up sample labels with directory names to find the correct output files from individual samples.
6. A number of results were generated from the cohort pipeline, including a master spreadsheet of somatic SNVs and INDELs that pass a bidirectionality filter (variants supported by reads from both strands), a read depth filter and a consequence filter (variants with deleterious consequences only). A plot was generated automatically, capturing mutation rates, mutational status of key melanoma-associated genes, as well as a breakdown of variant types (Figure S1). Copy number, transition/transversion, pathway, and clinical correlation analyses were all performed as part of the pipeline.

A number of key results of the study were derived from our automated pipeline. Correlation analysis against clinical annotation led to a few significant findings: The mutation rate in each melanoma sample was identified and found to vary widely between tumours, where melanomas arising in severely sun damaged skin have significantly higher mutation loads than non-severely sun damaged melanomas. *BRAF/NRAS* wild-type tumours were also found to have a higher average mutation rate compared to *BRAF/NRAS* mutant tumours. Furthermore, transition/transversion analysis led to a novel finding that tandem CC>TT/GG>AA mutations (UV damage signature) were more common in tumours arising in severely sun damaged skin and in *BRAF/NRAS* wild-type tumours. Pathway analysis suggested that potentially actionable mutations in wild-type tumours, including *NF1, KIT* and *NOTCH1*, were spread over various signalling pathways. Importantly, TREVA has been successful in the molecular subtyping of melanomas, which may direct novel therapeutic options for *BRAF/NRAS* wild-type patients.

#### Performance

Fastq files of the 34 tumour and the 34 matched blood samples (i.e. 68 whole-exome samples in total) were processed on a 64-bit Linux with 6 quad cores (24 CPUs) and 128GB RAM. The primary variant calling pipeline was run with a limit of 6 concurrent analyses (i.e. 12 samples) at any one time allowing up to 4 threads each. Analyses on all the 68 samples were completed in 9 days, with the most time-consuming steps being alignment, INDEL realignment and variant calling. The secondary pipeline for cohort analysis was run across all samples in a single run on 11 clinical variables. All clinical variables are processed in parallel by default. On the same server, the pipeline completed in 2 days, with the most time-consuming step being pathway analysis with Genomic MuSiC.

## Discussion: Versioning

Due to rapid evolvement in sequencing technologies and bioinformatics methods, it is often desirable to keep up-to-date with the latest release of the software packages that are used in a pipeline. With VMs, users would have the options to begin with a stable image, and then update individual packages as they wish. Installing an update may require updating other parts of the pipeline when, for example, there is a change in the input parameters or interface format requirements. In the case when an update breaks the pipeline, the original image can be easily restored (another benefit of the VM approach), avoiding update catastrophes where everything needs to be built from scratch.

Pipeline publishers should provide regular updates to their virtual images either via patches or brand new images. We are continually developing, testing and applying our pipelines and new versions will be made available as they become stable. We encourage other pipeline developers to publish their pipelines in the form of a Virtual Machine to enable the community to gain quick access to complex analyses.

An emerging tool called Vagrant [39], [40] is becoming popular in the software industry for building and configuring VMs with a focus on automation. We envisage this tool will further increase the value of using VMs in bioinformatics as it provides a systematic, lightweight way to update and deploy any analysis pipeline.

## Conclusion

We have proposed a novel solution to the problem of pipeline construction for TR/WES data analysis using a virtual appliance (TREVA), which requires minimal effort on the management and configuration of the underlying hardware and software systems. This allows TREVA to be transferrable to multiple laboratories or research institutions, enabling them to reproducibly run complex analysis pipelines with ease. TREVA is packaged with two types of analysis pipelines to cater for the analysis and interpretation of variations in the human and mouse genome, and to further allow for comparisons to be made between samples. TREVA is also streamlined for extension if required, enabling more complex pipelines to be built upon its original backbone. We envisage that the distribution of bioinformatics pipelines as virtual machines will be critical in the current era of big data, cloud computing, cheaper sequencing, and the need for faster and more efficient analysis of results.

## Supporting Information

Figure S1 **Plot generated automatically by the cohort pipeline for the example study of 34 primary cutaneous melanoma.**
(DOCX)

Table S1 Master variant call output file produced by the cohort pipeline.
(ZIP)

Table S2 Example “sample definition file” required by the cohort pipeline.
(XLSX)

Checklist S1 Feature comparison of bioinformatics pipelines.
(DOCX)
