# Supplementary material for: Bioinformatics Pipelines for Targeted Resequencing and Whole-Exome Sequencing of Human and Mouse Genomes: A Virtual Appliance Approach for Instant Deployment
Source: PLoS One. 2014 Apr 21;9(4):e95217. doi: 10.1371/journal.pone.0095217 (PMC3994043; doi:10.1371/journal.pone.0095217)
Supplement: Figure S1 — Plot generated automatically by the cohort pipeline for the example study of 34 primary cutaneous melanoma. (DOCX) [file pone.0095217.s001.docx]

# Supporting Information: Figure S1


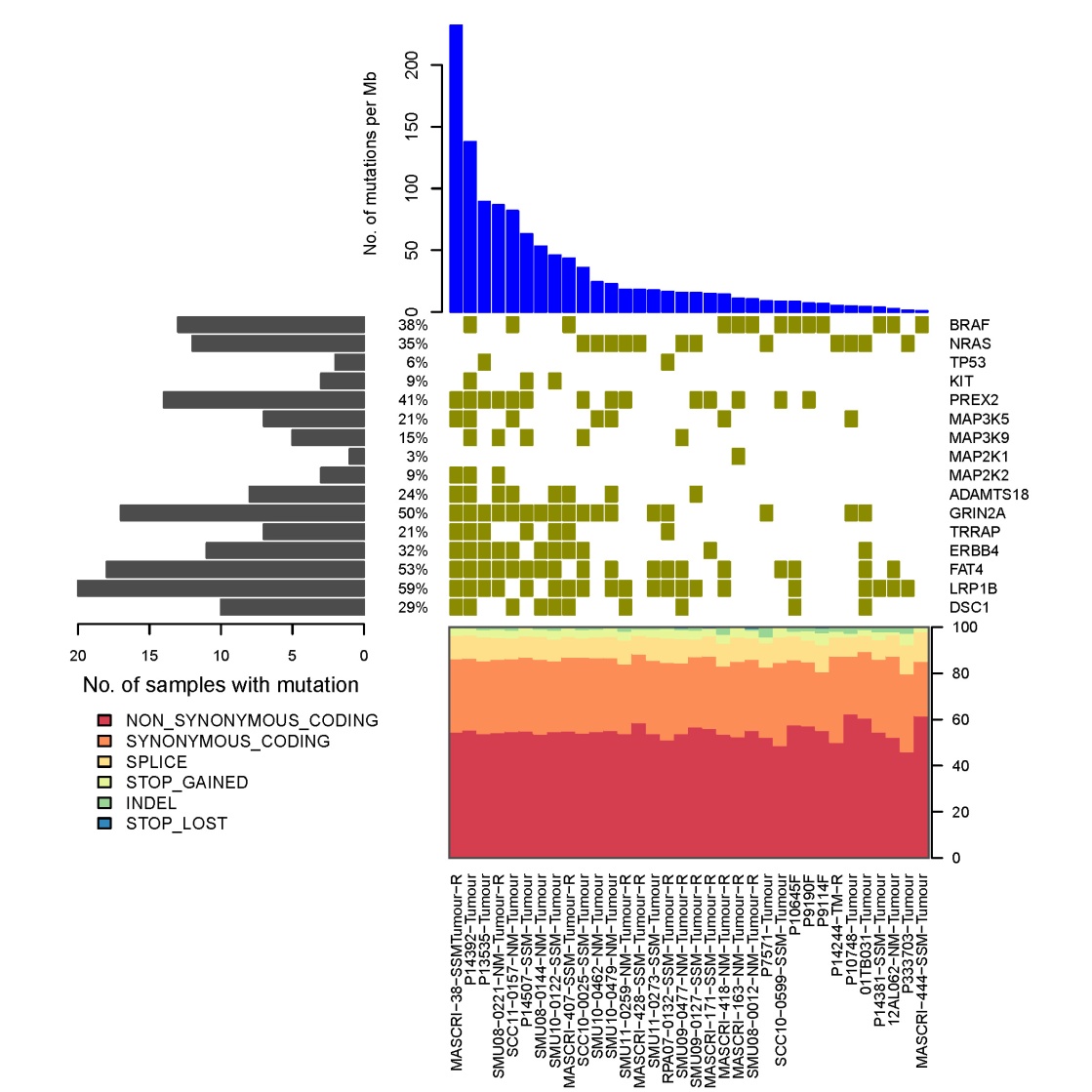


**Figure S1: Plot generated automatically by the cohort pipeline for the example study of 34 primary cutaneous melanoma.**
