# Supplementary material for: Bioinformatics Pipelines for Targeted Resequencing and Whole-Exome Sequencing of Human and Mouse Genomes: A Virtual Appliance Approach for Instant Deployment
Source: PLoS One. 2014 Apr 21;9(4):e95217. doi: 10.1371/journal.pone.0095217 (PMC3994043; doi:10.1371/journal.pone.0095217)
Supplement: Checklist S1 — Feature comparison of bioinformatics pipelines. (DOCX) [file pone.0095217.s004.docx]

**Checklist S1: Feature comparison of bioinformatics pipelines.**

|  | **TREVA** | **Galaxy** | **Atlas2 Suite** | **WEP resource** |
| --- | --- | --- | --- | --- |
| **Technical constraints** | Requires basic command line skills | - For web version, usage limited by data transfer rate and storage quota - Local version requires dedicated personnel with strong computing skills for setup and maintenance | Requires good command line and unix skills | Usage limited by data transfer rate and storage quota |
| **Installation** | Easy; based on VM | - Not required; available as a web service - Can install locally but difficult | Difficult; requires compilation and command line installation | Not required; available as a web service |
| **Usage** | - Command line - Pipelines can be started using a single command | - Web browser - Required to manually select individual analysis programs | - Command line - Required to call different modules separately | - Web browser |
| **Pipeline Scope** | Both single sample and multi-sample (cohort analysis) | Single sample | Single sample | Single sample |
| **Analysis** | - QC & trim - Read stats - Alignment - Duplicates removal - Realignment/ Recalibration - SNV calls - INDEL calls - CNV calls - Annotation Cross-samples significance and pathway analysis - Recurrent CNV analysis | - QC & trim - Read stats - Alignment - SNV calls - INDEL calls - Annotation   Can be customised to include more  Impractical to process >10 samples. | - QC & trim - Read stats - Alignment - Duplicates removal - SNV calls - INDEL calls - Annotation | - QC & trim - Read stats - Alignment - Duplicates removal - Realignment/ Recalibration - SNV calls - INDEL calls - Annotation |
| **Customisation** | Unix scripting | - Not available for web version - Local instance requires specialised scripting | Unix scripting | Not available |
